# Cell aging related genes can be used to characterize clinical prognoses and further stratify diffuse gliomas

**DOI:** 10.1038/s41598-021-98913-w

**Published:** 2021-09-30

**Authors:** Yang Yang, Xin Chen, Jianjun Sun, Suhua Chen, Chenlong Yang, Qianquan Ma, Jun Yang

**Affiliations:** 1 Department of Neurosurgery, Peking University Third Hospital, No.49 Huayuanbeilu, Haidian District, Beijing, 100191 China; 2 Center for Precision Neurosurgery and Oncology of Peking University Health Science Center, Beijing, 100191 China

**Keywords:** Tumour biomarkers, CNS cancer

## Abstract

Increasing evidence has indicated that senescent cells are associated with the glioma development. Thus, we aimed to explore the relationship between the cellular senescence gene profile and the clinical prognosis of diffuse glioma. In total, 699 gliomas from The Cancer Genome Atlas (TCGA) dataset were used as the training cohort and 693 gliomas from the Chinese Glioma Genome Atlas (CGGA) dataset were used as the validation cohort. Bioinformatics statistical methods are used to develop the risk signature and to study the prognostic value of the risk signature. We identified a 14-gene risk signature and its risk score was an independent prognostic factor (P < 0.001) in the validation dataset. The risk signature had better prognostic value than traditional factors for the 3- and 5-year survival rate. Importantly, the risk signature could further stratify gliomas in specific subgroups of World Health Organization (WHO) classification by the survival rate. Furthermore, the mRNA levels of genes involved in the cell cycle, cell division and other processes were significantly correlated with the risk score. Our study highlighted a 14-gene risk signature for further stratifying the outcomes of patients with gliomas with definite WHO subgroups. These results indicate the potential clinical implications of cell aging-related genes in gliomas.

## Introduction

Glioma is the most common primary malignant brain tumor in adults, with an incidence of 6–8 cases per 100,000 persons per year^1,2^. According to the latest WHO 2021 guidelines for glioma classification, the malignant degree of gliomas is graded from II–IV on the basis of morphological and molecular criteria^3^. Despite aggressive surgical resection followed by concomitant chemoradiotherapy and/or adjuvant chemotherapy, the prognosis of patients with high-grade tumors remains poor. Glioblastoma multiforme (GBM) is considered to be one of the most malignant primary intracranial tumors and has a dismal prognosis, the median overall survival (mOS) rate is approximately 15–18 months, and less than 10% of patients survive for more than 5 years^4–7^. Despite many efforts, most gliomas invariably recur and lead to a fatal outcome.

Cell aging, or cellular senescence, is believed to be a stress response triggered by many "counting mechanisms" that have been increasingly understood at the molecular level. Importantly, when cell senescence is accompanied by certain gene mutations, it is carcinogenic. In humans, senescent cells were identified in benign lesions of the skin carrying oncogenic mutant BRAF^8^ and in neurofibromas from patients with NF1 mutations^9^. In short, there is now convincing evidence that senescent cells are associated with the precancerous stage of tumor development. On the other hand, most of the current studies on the relationship between cellular senescence and tumorigenesis are focused on tumors elsewhere in the body, and the roles of cell aging factors in glioma occurrence and development are rarely studied. Researchers such as Coppola et al. found senescence-associated genes linked to age, prognosis, and progression of human gliomas^10^. However, it is believed that the role of senescent cells in the microenvironment of glioblastoma after radiotherapy may have dual effects^11–13^. Some researchers found radiation delays recurrence by inducing cellular senescence^12^, the other found radiation-induced DNA damage leads to the senescence of nontumor cells in the microenvironment, which may lead to tumorigenesis and recurrence^14,15^. Considering that the abnormal expression of cellular senescence-related genes may lead to changes in the RNA expression profile of gliomas, it is of great significance to systematically study the role of cell aging-related genes in glioma.

To better understand the influence of cellular senescence-related genes on the prognosis of gliomas, we systemically analyzed the RNA expression profile of cell aging-related genes and their prognostic values in gliomas from the TCGA (n = 631) and the CGGA (n = 693) database. We also identify a panel of 14 cell aging-related genes as a risk signature, and a functional analysis of the genes that correlated with the signature risk score in glioma was performed.

## Results

### Prognostic value of cell aging-related genes and their biological function in gliomas

**Figure 1:**
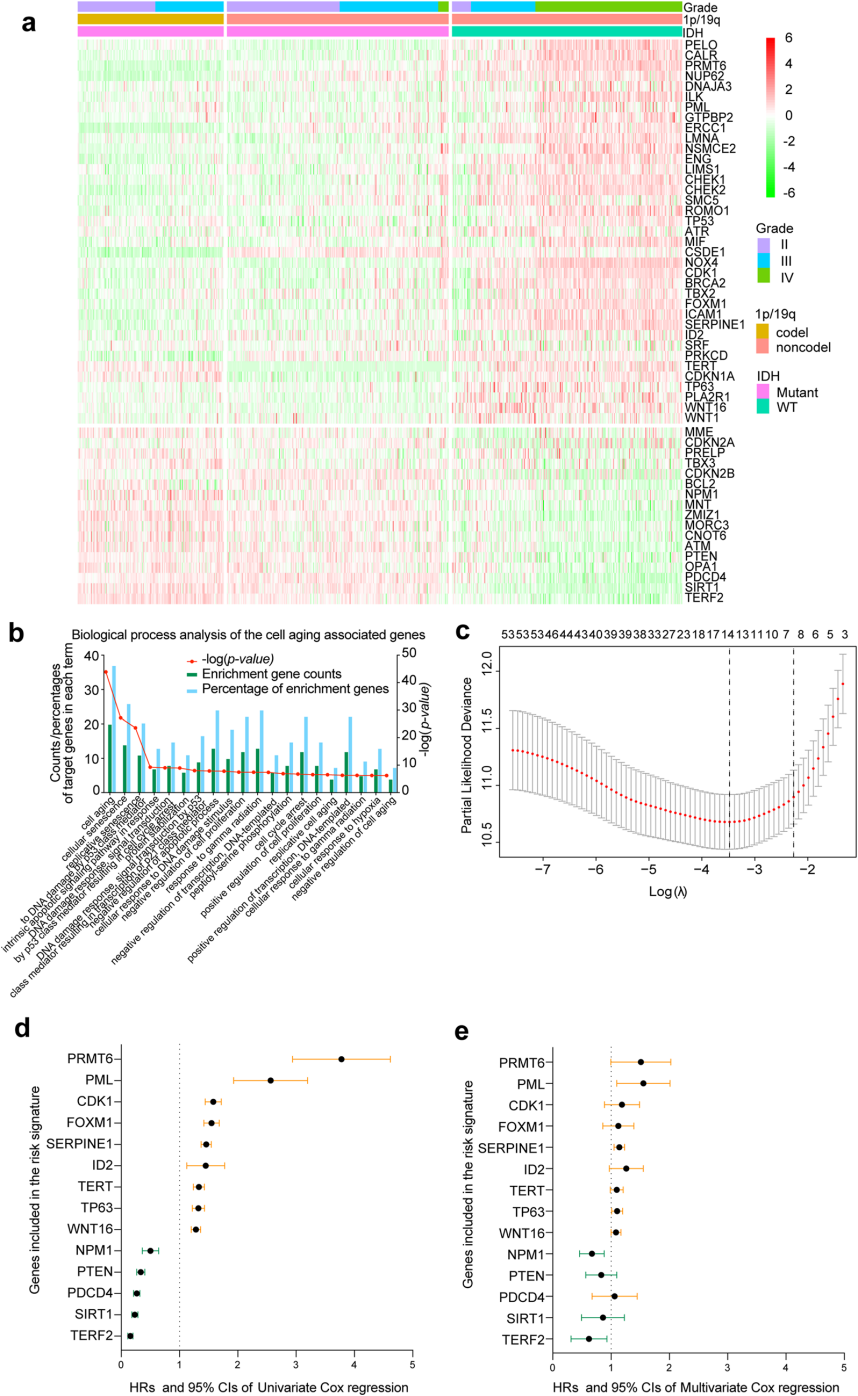
Prognosis-associated cell aging-related gene expression profile in gliomas. (**a**) Heatmap showing the expression pattern of the 54-cell aging-related genes associated with patient survival. (**b**) GO biological process terms enriched among the 54 survival-associated cell aging-related genes. (**c**) The 14-gene risk signature calculated by using LASSO. (**d**,**e**) The 14 genes included in the signature, their HRs, and 95% CIs by univariate Cox regression analysis and multivariate Cox regression analysis.

Of the 102-cell aging-related genes tested, 68 genes with available RNA expression data in the TCGA data sets were used for further analysis. Among these genes, 54 were significantly correlated with the OS rate of patients with glioma. A total of 17 of the 54 genes had a hazard ratio (HR) < 1 and were considered protective factors, while the remaining 37 genes had an HR > 1 and were considered risk factors. Furthermore, risk-associated genes with increased expression and protection-associated genes with decreased expression were associated with increased malignancy in 3 subgroups according to the WHO 2016 classification of central nervous system tumors (Fig. 1a). Some of these genes also showed inconsistent expression levels in the same subgroup, which suggested the potential prognostic value of these genes for a specific subgroup.

These 54 genes are a collection of genes that participate in any process involved in the gradual loss of cellular functions and eventually result in cell death. We used Gene Ontology (GO) analysis to study the more specific biological processes that these genes are enriched in, and the results indicated that these survival-associated genes were correlated with GO terms such as cell aging, cellular senescence, replicative senescence, intrinsic apoptotic signaling pathway in response to DNA damage by p53 class mediator, DNA damage response, signal transduction by p53 class mediator resulting in cell cycle arrest, DNA damage response, signal transduction by p53 class mediator resulting in transcription of p21 class mediator and other cellular senescence-related terms (Fig. 1b).

### Identification of a panel of 14 cell aging-related genes as a risk signature in glioma

To easily and reliably stratify the outcomes of patients with glioma using cell aging-related gene expression data, we applied the LASSO Cox regression algorithm to the 54-cell aging-related genes in the TCGA data set (Fig. 1c). A total of 14 genes were selected to build the risk signature, and the coefficient and normalized expression levels of these genes were used together to calculate risk scores for the TCGA data set. The 14 genes included in the signature, their HRs, and 95% confidence intervals (CIs) by univariate Cox regression and multivariate Cox regression analysis were calculated (Fig. 1d,e). The results indicated that *PRMT6, PML, CDK1, FOXM1, SERPINE1, ID2, TERT, TP63* and *WNT16* had HRs > 1 and that *NPM1, PTEN, PDCD4, SIRT1* and *TERF2* had HRs < 1, similar to the results of multivariate Cox regression analysis, which showed that the results were stable and reliable.

To investigate the prognostic value of the risk signature and other clinicopathological characteristics, univariate and multivariate Cox regression analyses were performed of both the TCGA (training set) and the CGGA (validation set) data sets. The results showed that a high-risk score was a prognostic factor for both data sets (P < 0.001), independent of WHO grade, age and sex (Table 1).

**Table 1:** Univariate and multivariate Cox regression in the TCGA and CGGA data sets.

		Univariate Cox analysis				Multivariate Cox analysis			
		P value	HR	HR (95% CI)		P value	HR	HR (95% CI)	
				Upper	Lower			Upper	Lower
TCGA data set	WHO grade	< 2E−16	4.8759	6.176	3.85	8.16E−09	2.344407	3.132	1.7548
	Age	< 2E−16	1.07336	1.085	1.062	3.08E−11	1.043787	1.057	1.0307
	Sex	0.965	0.99387	1.313	0.7524	0.856	1.026288	1.358	0.7753
	Risk score (high vs. low)	< 2E−16	1.21757	1.247	1.189	3.46E−07	1.096659	1.136	1.0584
CGGA data set	WHO grade	< 2E−16	3.42574	4.126	2.844	< 2e−16	2.4242	2.981	1.9711
	Age	2.65E−09	1.03037	1.041	1.02	0.122	1.007195	1.016	0.9981
	Sex	0.909	1.01398	1.286	0.7992	0.286	1.140823	1.453	0.8955
	Risk score (high vs. low)	< 2E−16	1.54265	1.627	1.463	< 2e−16	1.356951	1.458	1.2629

P value obtained by univariate and multivariate Cox regression analysis.

In order to analyze the expression characteristics of 14 genes in senescent cells and proliferating cells, we consulted the literature and learned about the Cell Age database (https://genomics.senescence.info/cells/query.php), which is a database of genes associated with cell senescence in different human cell types. Our analysis results are presented in Figure S1, 10 of 14 genes were found in the database. We found that whether it is genes with HR > 1 or HR < 1, the effects on cell senescence are inconsistent. In order to analyze the expression characteristics of 14 genes in tumors as compared to normal tissues, we searched the TCGA database for the expression of these 14 genes in 31 tumors and their normal control tissue (Figure S2), and we found PRMT6, PML, CDK1, FOXM1, SERPINE1, ID2, TP63 and NPM1 was significantly overexpression in GBM (Figure S3).

### The risk signature predicted unfavorable prognosis of patients with glioma

**Figure 2:**
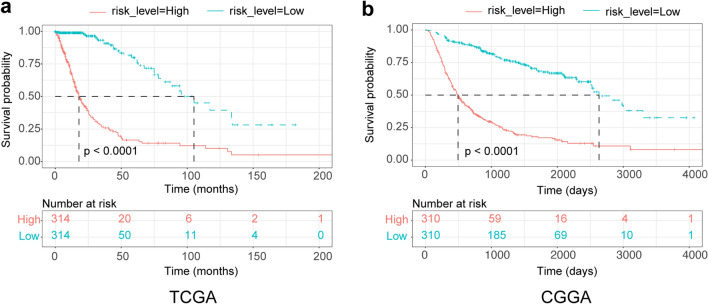
Outcome prediction for the patients in the TCGA and CGGA cohorts. (**a**) Kaplan–Meier survival curves of the high-risk and low-risk groups in all gliomas evaluated in the TCGA data set. (**b**) Kaplan–Meier survival curves of the high-risk and low-risk groups in all gliomas evaluated in the CGGA data set.

To further analyze the prognostic value of the risk score of the 14-gene signature, we employed dichotomization according to the cutoff value to separate glioma patients for the Kaplan–Meier survival analysis. We divided patients into high-risk and low-risk groups using the respective median risk score of various stratified glioma subtypes as the cutoff value. We found that patients with low-risk scores had significantly longer OS rates than patients with high-risk scores among all gliomas evaluated from the TCGA data sets (Fig. 2a, P < 0.0001). Similar results were shown for the CGGA dataset, in which the risk score was a biomarker of poor prognosis in glioma patients (*P* < 0.0001, Fig. 2b).

### Validation of the prognostic value of the 14-gene signature

**Figure 3:**
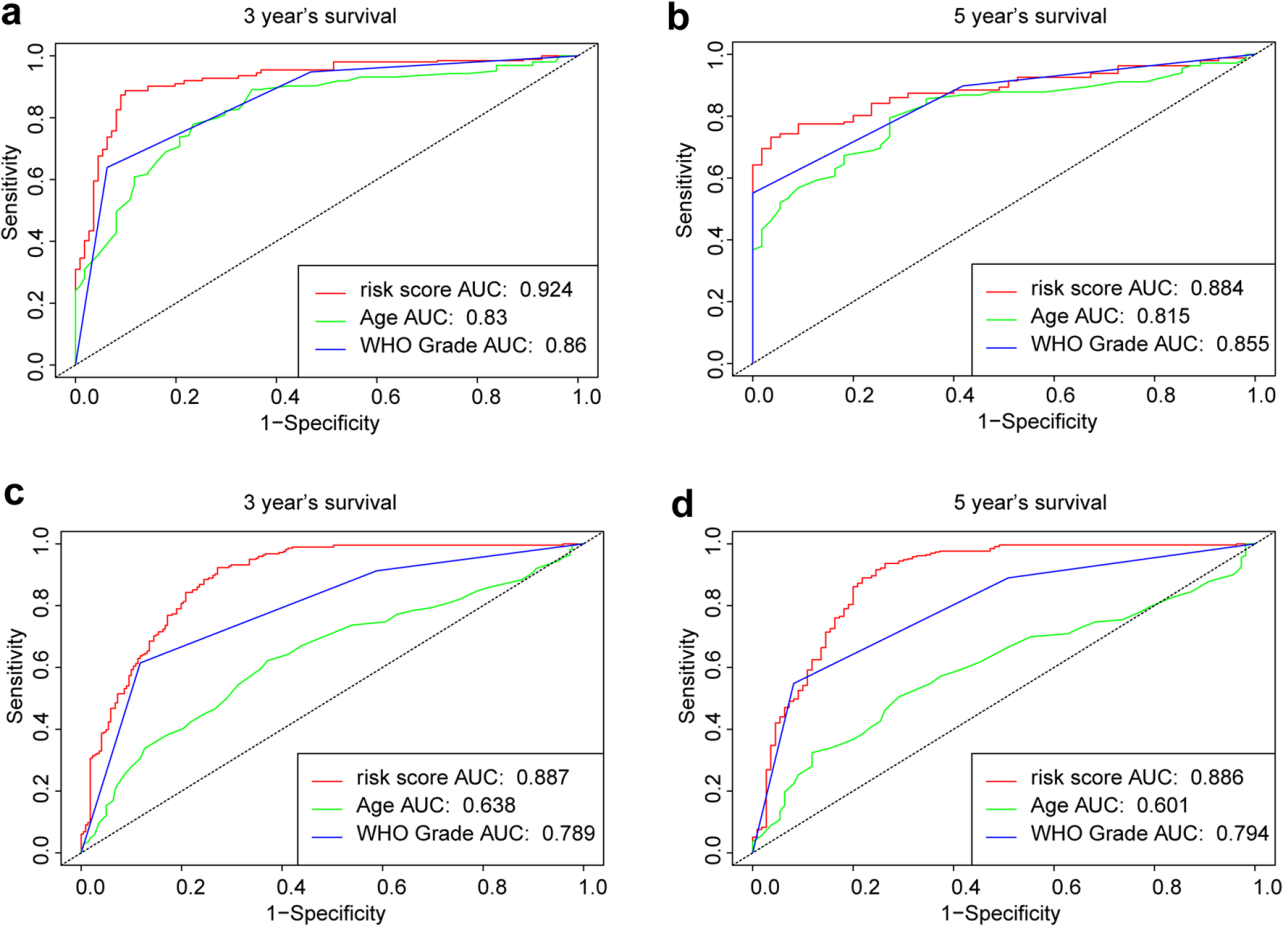
Comparisons of survival predictive efficiencies between the risk score and clinicopathological characteristics. ROC curves showed the predictive efficiencies of risk scores, age and WHO grade on 3-year and 5-year survival rates in the TCGA (**a**,**b**) and CGGA (**c**,**d**) data sets.

To evaluate the prediction accuracy of the risk score for the OS rate, receiver operating characteristic (ROC) curve analysis was performed. The results showed that the signature risk score had the best efficiency (compared with age and WHO grade) for predicting the 3-year and 5-year survival rates of patients with gliomas from both the TCGA and CGGA data sets (Fig. 3). The area under the curve of risk score, age, and WHO grade were 92.4%, 83% and 86%, respectively, for the 3-year survival rate in the TCGA data set; 88.4%, 81.5% and 85.5%, respectively, for the 5-year survival rate in the TCGA data set; 88.7%, 63.8% and 78.9%, respectively, for the 3-year survival rate in the CGGA data set; and 88.6%, 60.1% and 79.4%, respectively, for the 5-year survival rate in the CGGA data set.

### The cell aging-related signature is associated with pathologic features of gliomas

**Figure 4:**
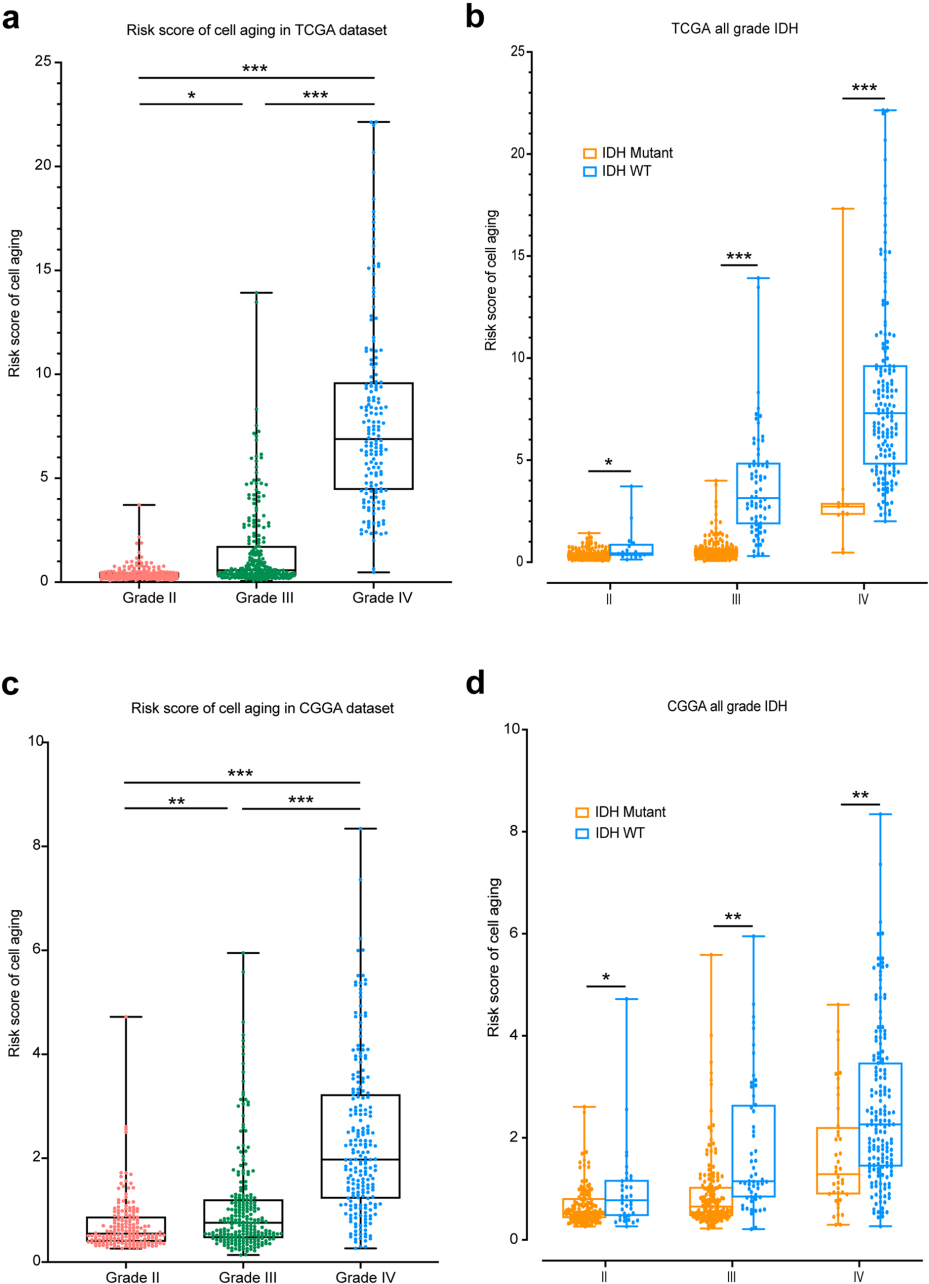
Risk score of the signature and relationship between risk score and IDH mutation in the TCGA and CGGA data sets. Risk score of the signature in the TCGA (**a**) and CGGA (**c**) data sets according to WHO grade. Relationship between risk score and IDH mutation in the TCGA (**b**) and CGGA (**d**) data sets.

We investigated the 14-gene signature risk score across glioma grades and subtypes defined by expression clusters by the TCGA workgroup. As expected, a higher risk score was correlated with a higher tumor grade, which indicated a malignant biological property of the risk genes in the TCGA cohort (Fig. 4a). To validate our findings, the 14-gene risk signature was also evaluated using the CGGA data set, and similar results were obtained (Fig. 4c). Isocitrate dehydrogenase mutation has been widely acknowledged as the earliest genetic alteration in glioma development. To investigate the influence that IDH may exert on the risk score, we analyzed the risk score of the 14-gene signature in IDH‐mutant type and wild‐type glioma. In the TCGA dataset, when taking grade into account, a significant difference was observed in grade II, III and IV gliomas (Fig. 4b). Similarly, in the CGGA dataset, the risk score was profoundly increased in the IDH‐mutant type of grade II, III and IV gliomas (Fig. 4d).

### Functional analysis of genes associated with cell aging that correlated with the signature risk score in gliomas

**Figure 5:**
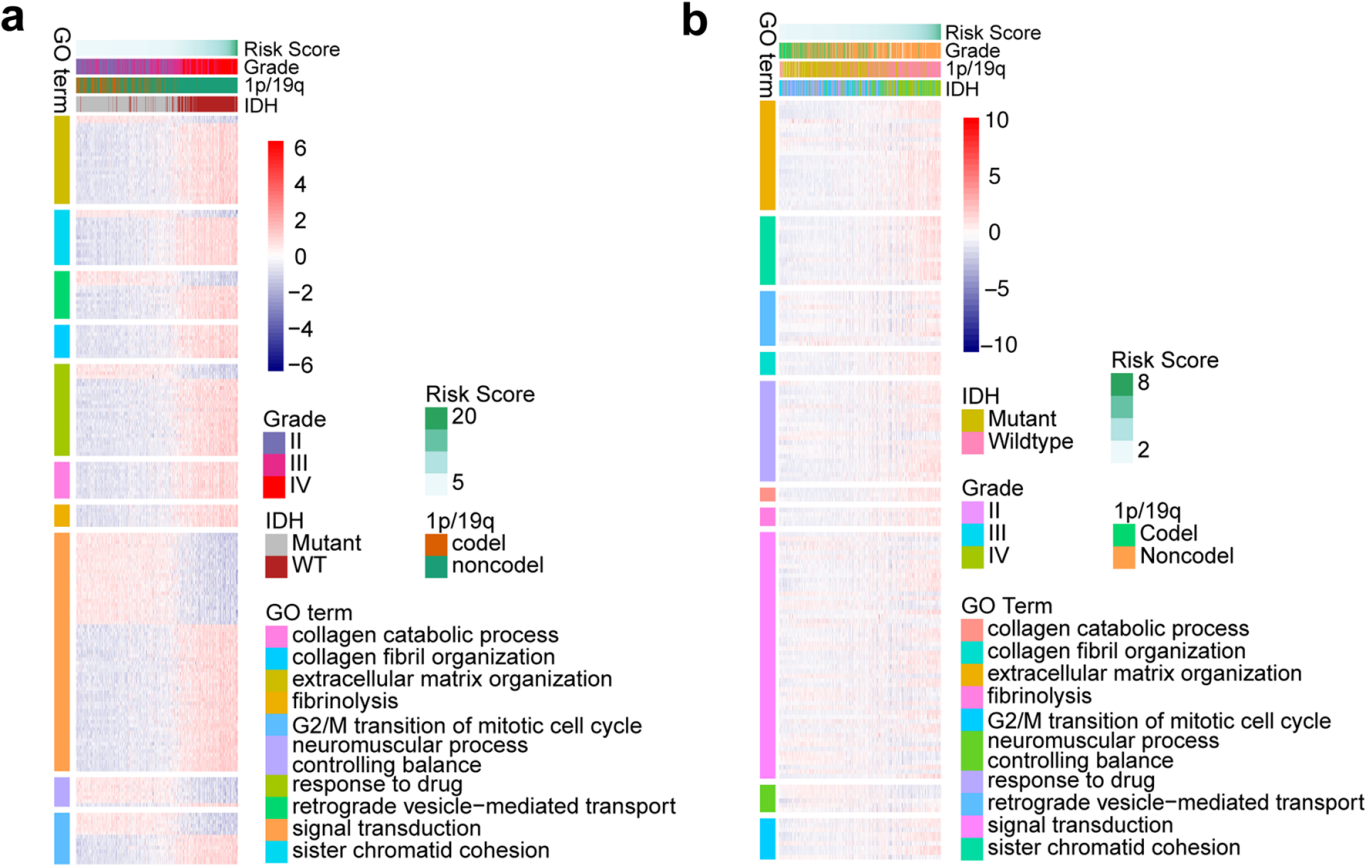
The biological functions of cell aging-related genes in the TCGA (**a**) and CGGA (**b**) cohorts.

To further investigate the biological significance and explain the results above, we utilized Pearson correlation analysis to select genes that were strongly correlated with the risk score (*Pearson* R ≥ 0.6 in the CGGA and TCGA data sets). A total of 631 and 693 genes in the TCGA and CGGA datasets met the criteria, respectively. To yield an accurate analysis, the differentially expressed genes that were shared between the two datasets (189 genes) were selected for Gene Ontology analysis with DAVID (https://david.ncifcrf.gov/). We found that the identified genes were typically correlated with biological functions of collagen catabolic process, collagen fibril organization, extracellular matrix organization, fibrinolysis, G2/M transition of mitotic cell cycle, neuromuscular process, response to drug, retrograde vesicle-mediated transport, signal transduction and sister chromatid cohesion. (Fig. 5a,b). Most of the terms identified were related to the cell cycle and cell division, partially explaining the increased malignancy of tumors and poor survival of patients in the high-risk score group.

**Figure 6:**
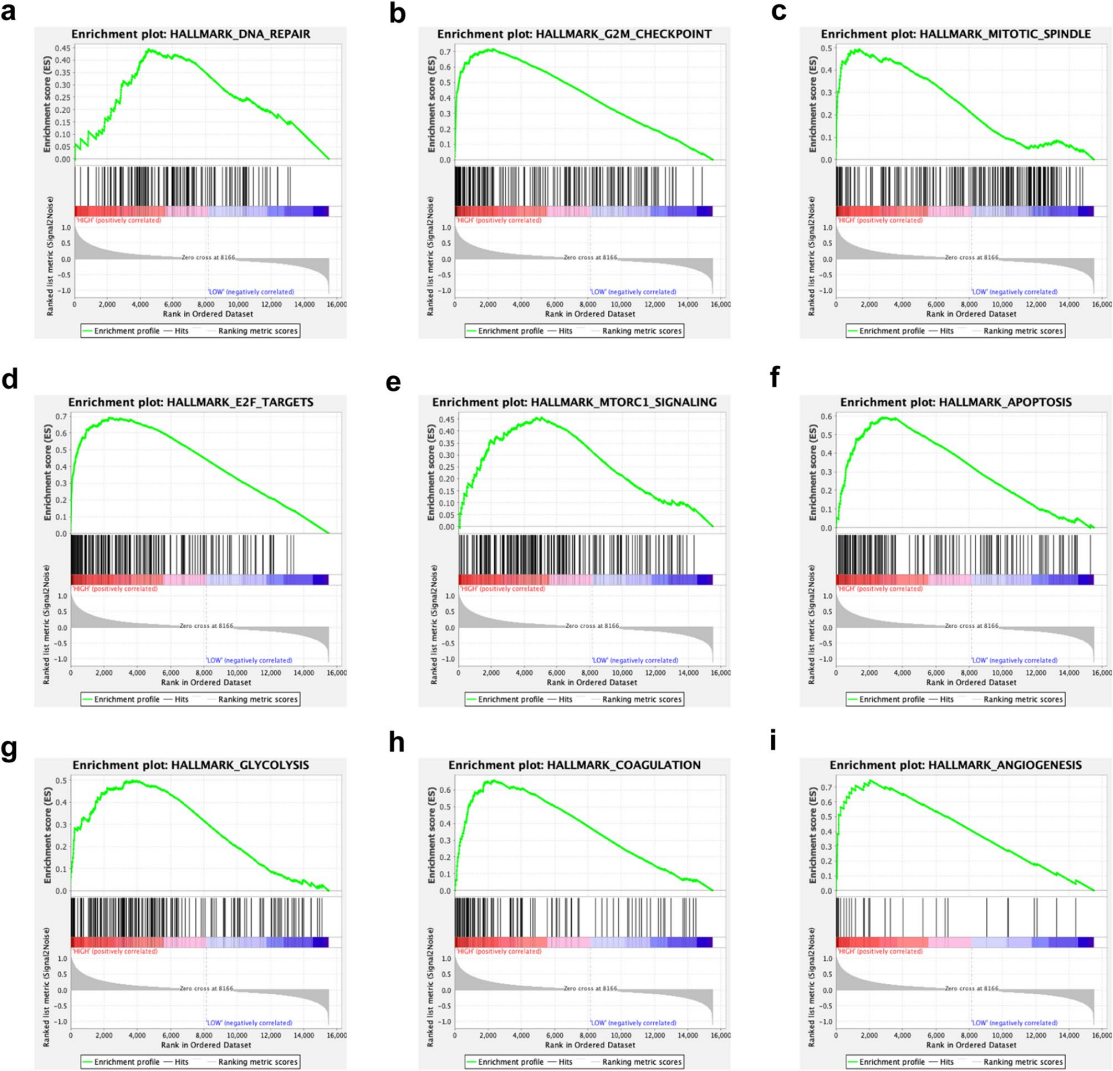
Functional analysis of the 14-gene signature. (**a**–**i**) GSEA revealed the hallmarks of malignant tumors, including DNA repair, G2/M checkpoints, mitotic spindles, E2F targets, MTORC1 signaling, apoptosis, glycolysis, coagulation and angiogenesis.

Furthermore, gene set enrichment analysis (GSEA) revealed the hallmarks of malignant tumors, including DNA repair, G2/M checkpoints, mitotic spindles, E2F targets, MTORC1 signaling, apoptosis, glycolysis, coagulation and angiogenesis (Fig. 6). These findings indicate that the risk score of the 14-gene signature reflects the expression alterations of genes involved in malignant biological processes, signaling pathways, and malignant hallmarks in gliomas, which might contribute to the patients’ high risk and poor prognosis.

## Discussion

In this study, we found that the general expression pattern of cell aging-related genes is correlated with the malignancy features of gliomas and identified cell aging-related genes that significantly participate in many processes involved in the gradual loss of cellular functions and cell death, which is associated with glioma prognosis. We further built a 14-gene risk signature that not only precisely predicted the unfavorable prognosis of gliomas more effectively than the WHO grade but also reflected malignancy-correlated clinicopathological features, such as IDH mutation status and WHO grade. Moreover, we systemically analyzed the function of genes that correlated with the signature risk score in gliomas and identified the corresponding functions, biological processes, key signaling pathways, and hallmarks, which might partially explain the malignancy of gliomas and the poor survival of patients in the high-risk group. Our results highlight the role of cell aging-related genes in the survival of patients with further stratified gliomas, and the 14-gene signature has the potential to be a more effective for determining the prognosis of gliomas with further stratification according to the WHO 2016 integrated diagnostic criteria. Furthermore, we also revealed the biological functions of the genes that were associated with risk signature.

The current mainstream view is that cell aging likely promotes glioma occurrence and development because glioma is more common in the elderly, and the median age at diagnosis is ≥ 60 years. At this age, the number of senescent cells is greatly increased in the brain. Moreover, cell aging in gliomas is likely result in posttreatment recurrence. There are two theories as to why cell aging may be the driving factor of residual disease. (A) The combination of temozolomide (TMZ) and radiation therapy, which is usually performed after surgery, induces cell aging in the microenvironment surrounding the tumor. Researchers have observed the presence of aging cells in glioma samples, especially in the tumor microenvironment, where residual glioma cells are also located. Ionizing radiation can cause brain aging in mice, which is believed to cause abnormal function^16^, and TMZ causes senescence in astrocytes in vitro^17^. Therefore, DNA-damaging antitumor therapies have been shown to induce senescence in healthy cells. (B) Aging brain cells produce and secrete a large number of factors, from extracellular matrix (ECM) components to remodeling factors, some of which can promote tumor survival and invasion. For example, astrocytes from aging primate brains synthesize excess hyaluronic acid, supporting single-cell invasion and NF-κB activation^18^; aging astrocytes also produce excessive levels of fibronectin, which may promote cell survival and collective invasion^19^; and aging astrocytes express both MMP-2 and MMP-9, which are necessary factors glioma invasion of multiple brain regions^20^. It is well known that aging astrocytes and endothelial cells also secrete a large number of cytokines and chemokines. Among them, IL6 independently correlated with either age, prognosis, or the grade of the glioma, and activates STAT3, which is a reciprocally regulated cytokine network in tumor cells, including inflammation and angiogenesis factors such as IL-8^10^.

It has been found that conditioned medium from aging astrocytes in vitro can promote the proliferation of glioma cell lines by increasing c-Myc; more importantly, this conditioned medium can improve the survival of TMZ-treated glioma cells^21^. Additionally, although residual glioma cells are very different from the original tumor, they secrete factors that are part of the aging-associated secretory phenotype (SASP), which maintains residual gliomas through therapy and drives drug resistance and recurrence^22^. Altogether, these results support the concept that the senescence-related secretory phenotype is related to the induction and age-related progression of glioma.

We identified a 14-gene risk signature related to cell aging in gliomas. The fourteen genes included in our signature were *PRMT6, PML, CDK1, FOXM1, SERPINE1, ID2, TERT, TP63*, *WNT16*, *NPM1, PTEN, PDCD4, SIRT1* and *TERF2*. Among them, nine genes included in our signature were associated with high risk, and patients with high expression of these genes had a poor prognosis. The remaining five genes were associated with low risk. As shown below, nine genes have similar functions and are involved in cell mitosis, proliferation, metastasis and progression of glioma. Some studies found that PRMT6 methylation of the RCC1 signaling axis regulates mitosis, tumorigenicity, and the radiation response of glioblastoma stem cells^23^. Furthermore, the PML/Slit axis controls physiological cell migration and cancer invasion in gliomas^24^. Studies have suggested that blockade of CDK1 induces synthetic lethality in malignant gliomas^25^; moreover, FoxM1 promotes β-catenin nuclear localization and controls Wnt target gene expression and glioma tumorigenesis^26^; ID2 promotes the survival of glioblastoma cells during metabolic stress by regulating mitochondrial function^27^. A multicenter study revealed that methylation of the TERT promoter in childhood gliomas could be used for risk stratification^28^ and NPM1 histone chaperone expression is upregulated in glioblastoma to promote cell survival and maintain nucleolar shape^29^. On the other hand, another four genes have functions involved in glioma suppression. Loss of tumor suppressor PTEN function increases immune resistance in gliomas^30^. Researchers found that downregulation of Pdcd4 facilitates glioblastoma proliferation in vivo^31^, the SIRT1 activator SRT2183 suppresses glioma cell growth^32^, and molecular targeting of TRF2 suppresses the growth and tumorigenesis of glioblastoma stem cells^33^.

In this study, the risk score of patients reflects the 14-gene expression patten of the signature combined with the survival status and survival time of each patient. Therefore, risk score act as a reflection of the prognosis of each patient. The risk score reflects the overall effect of 14 genes in signature on the overall survival of patients, not determined by the expression of a single gene. Moreover, the purpose of this study is to clarify the relationship between the predictive power of the signature and outcome. In senescent cells and proliferating cells, we found that whether it is genes with HR > 1 or HR < 1, the effects on cell senescence are inconsistent. Some of the risk factors inhibit aging, some promote aging, and the same is true for protective factors. Regulation patterns of protection and risk factors cannot be determined in senescent cells vs proliferating cells. Because the protection and risk factors are for tumor patients, and the relationship between senescent cells and tumors is very complicated. Some researchers found in the early stage of tumor, cell senescence inhibits tumor growth, and in the late stage of tumor senescence promotes tumor progression^34^.This is consistent with the theory that aging is a double-edged sword in tumors^35^. Therefore, the role of cell senescence in senescent cells and proliferating cells is worthy of further study.

Moreover, protective factors and risk factors have different regulation modes in tumors vs normal tissues. Protective factors inhibit tumor progression, risk factors promote tumor progression and shorten patients’ survival. And in this study, risk score reflects the overall effect of 14 genes in signature on the overall survival of patients. This includes the effect of protective factors with HR < 1 and risk factors with HR > 1, which is the effect of combining the two factors to predict the overall survival of patients. We found that some researchers believe that the effect of cellular senescence on tumors depends on the stage of tumor progression^34^, other researchers believe that aging has a dual effect on tumors^36^. So, aging has a dual effect on the regulation pattern of tumors, which may depend on the stage of tumor progression and the degree of malignancy.

In summary, our study highlighted the strong clinical prognostic value of cell aging-related genes in gliomas and revealed a risk signature with 14 cell aging-related genes to further stratify the outcomes of patient with gliomas with definitive WHO subgroups. Clinical characteristics, pathological features, biological processes, significant signaling pathways and hallmarks of gliomas correlated with the risk signature were also identified. These results provide fundamental information for understanding the roles of cellular senescence in gliomas and indicate the potential clinical implications of cell aging-related genes in diffuse gliomas.

## Conclusion

Using the TCGA dataset as the training cohort and the CGGA dataset as the validation cohort, a 14-gene risk signature of cell aging-related genes was identified through integrated bioinformatic analysis, and the prognostic value of the risk signature was validated. Furthermore, the cell aging-related signature is associated with the pathologic features of glioma, and the risk signature could further stratify the survival of patients with gliomas in specific subgroups of the WHO classification. Finally, GO and signaling pathway enrichment analysis showed that the mRNA expression levels of genes involved in the cell cycle, cell division and other processes were significantly correlated with risk scores. These findings could significantly enhance our understanding of the roles of cellular senescence in glioma and indicate the potential clinical implications of cell aging-related genes in glioma.

## Material and methods

### Patients

The data from a total of 699 glioma samples were collected from the TCGA database (http://cancergenome.nih.gov). Of these TCGA samples, we excluded 68 samples that did not have available RNA-seq data, molecular pathological information or useful OS information; the other 631 glioma samples with RNA-seq transcriptome data and corresponding clinical and molecular pathological information available were obtained from the TCGA and used for systematic analysis. Another 693 glioma samples with RNA-seq transcriptome and corresponding clinicopathological information available were obtained from the CGGA database (http://www.cgga.org.cn/) and were used to validate the performance of the risk signature^37^.

### Selection of cell aging-related genes

We first collated a list of 102 genes that participated in any process involved in cell aging (http://amigo.geneontology.org/amigo/term/GO:0007569). Then, we used the 68 genes with available RNA expression data from the TCGA data set for further analysis.

### Identification of the risk signature

We performed univariate Cox regression analyses of the expression of RNA processing genes to identify genes with expression that was significantly correlated with the prognosis of patients with gliomas from the TCGA data set. Next, we used the LASSO Cox regression algorithm to build an optimal risk signature with the minimum number of genes. Finally, a set of genes and their coefficients were determined by minimum criteria and validated by multivariate Cox regression analyses.

### Gene ontology (GO) and genes and genomes (KEGG) pathway enrichment analysis

The functional enrichment of the genes was assessed based on GO terms and KEGG pathway annotations. GO term analyses were performed using the DAVID database (https://david.ncifcrf.gov/), which is an essential tool for the success of high‑throughput gene function analysis. Pathway analysis was also conducted using the online DAVID database. P‑values of < 0.05 were considered to denote statistically significant differences in GO term enrichment and KEGG pathway analyses, and the false discovery rate was utilized to correct the *P*‑values.

### Statistical analysis

Patients were divided into high-risk and low-risk groups using the median risk score as the cutoff value. Patients could also be compared between groups with lower risk scores and higher risk scores. The Kaplan–Meier method with a 2-sided log-rank test was used to compare the OS rate of patients in the high-and low-risk groups. All statistical analyses were conducted using R (https://www.r-project.org/) and Prism 8 (GraphPad Software Inc.). Univariate and multivariate Cox regression analyses were performed to determine the prognostic value of the risk score and various clinical and molecular pathological characteristics. ROC curve analysis was used to predict OS rates with the R package “pROC”. GSEA was performed to identify gene sets with significant differences between two groups by using GSEA v4.1.0 software (http://www.gsea-msigdb.org/gsea/index.jsp).

## Supplementary Information

Supplementary Information.
