# Supplementary material for: Cell aging related genes can be used to characterize clinical prognoses and further stratify diffuse gliomas
Source: Sci Rep. 2021 Sep 30;11:19493. doi: 10.1038/s41598-021-98913-w (PMC8484278; doi:10.1038/s41598-021-98913-w)
Supplement: Supplementary file 1 — Supplementary Information. [file 41598_2021_98913_MOESM1_ESM.pdf]

Supplementary Materials

Gene Symbol	Method	Cell Types	Cell Lines	Cancer Line?	Senescence Type	Senescence Effect
PRMT6	Knockdown	Breast cancer, Breast epithelial	MCF-10A, MCF-7, MDA-MB-231, MDA-MB-468, SK-BR-3	Yes	Replicative	Inhibits
PML	Overexpression	Lung fibroblast	WI-38	No	Oncogene-induced	Induces
CDK1	Knockdown	Embryonic kidney, Retinal pigment epithelial	HEK293T, hTERT-RPE1	Yes	Replicative	Inhibits
FOXM1	Knockdown	Gallbladder cancer	GBC-SD	Yes	Replicative	Inhibits
SERPINE1	Knockdown	Foreskin fibroblast	BJ, Primary cell	No	Replicative	Induces
TERT	Overexpression	Foreskin fibroblast, Retinal pigment epithelial	BJ, RPE-340	No	Replicative	Inhibits
TP63	Knockout	Foreskin fibroblast	BJ	No	Replicative	Induces
WNT16	Knockdown	Lung fibroblast	MRC-5	No	Replicative	Induces
SIRT1	Overexpression	Lung fibroblast	2BS	No	Replicative	Inhibits
TERF2	Knockout	Neuroblastoma	SH-SY5Y	Yes	Replicative	Inhibits

Figure S1. The expression characteristics of 14 genes in senescent cells and proliferating cells. 10 of 14 genes were found in the database. Red indicates genes with HR>1, and green indicates genes with HR<1.

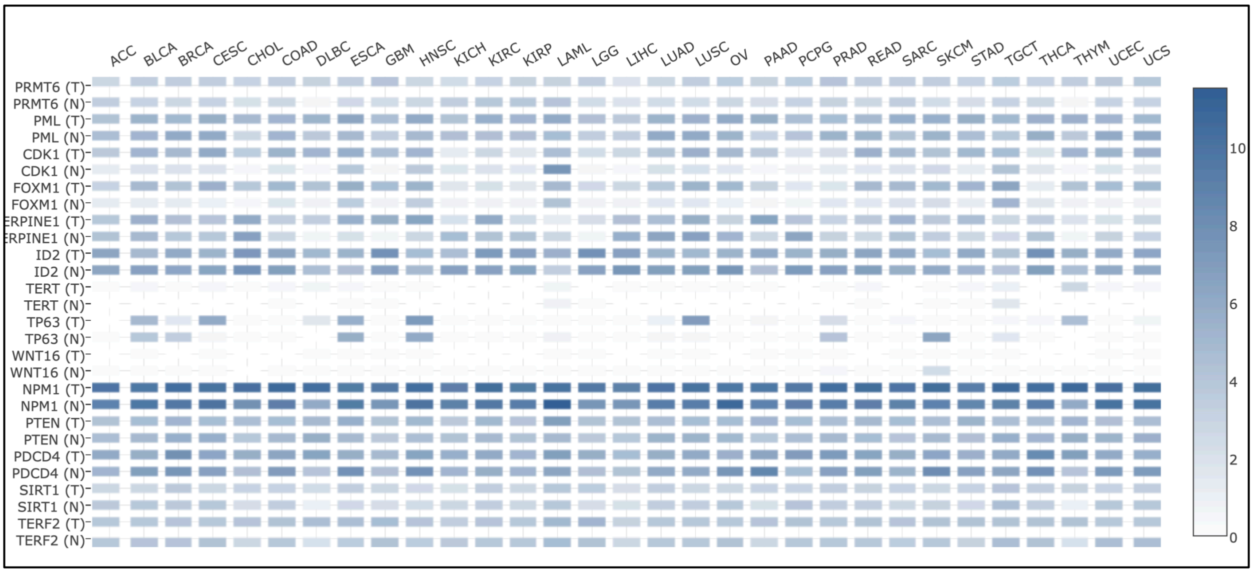

Figure S2. The expression of these 14 genes in 31 tumors and their normal control tissues

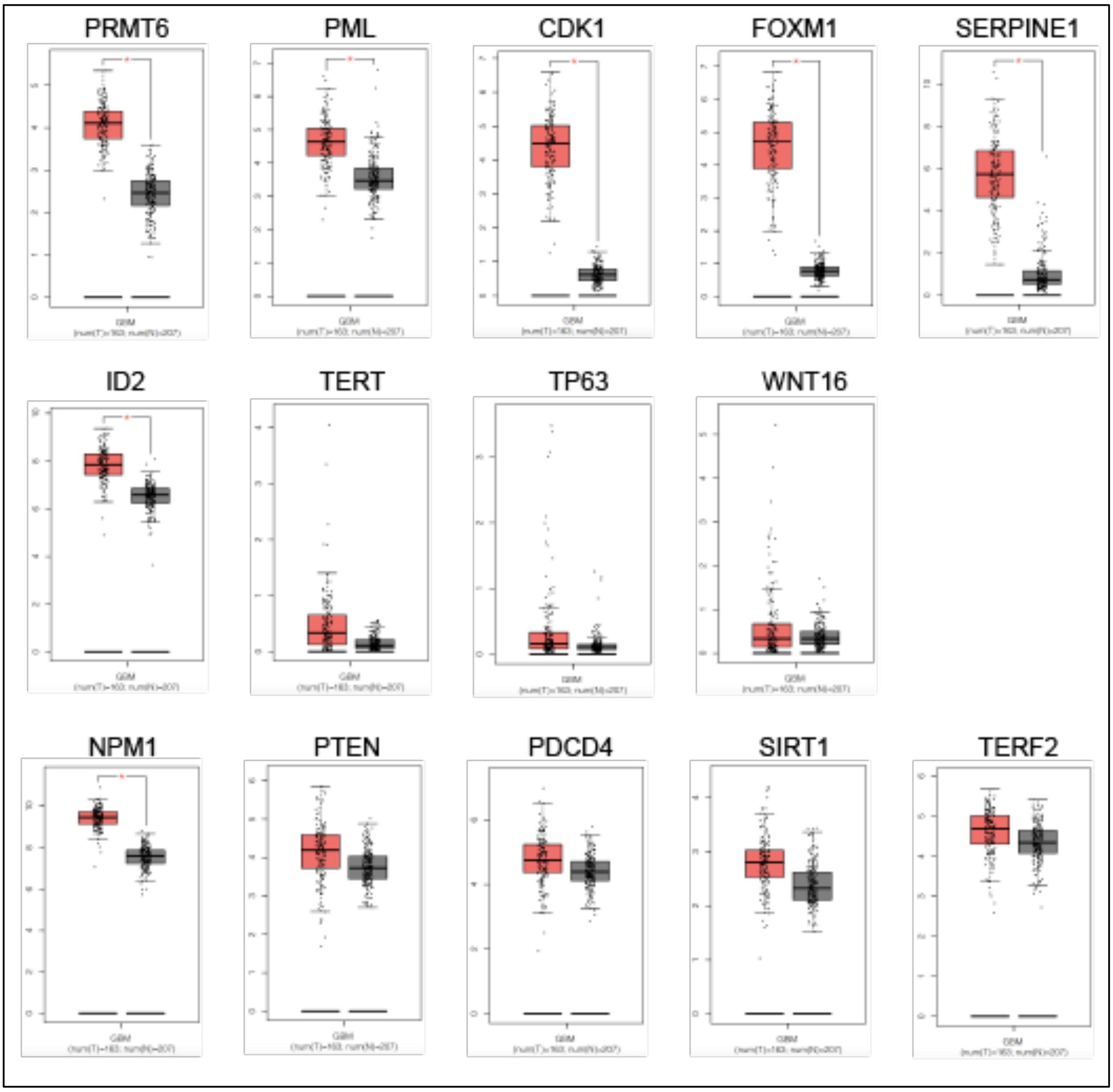

Figure S3. The expression of these 14 genes in GBM in TCGA database. PRMT6, PML, CDK1, FOXM1, SERPINE1, ID2, TP63 and NPM1 was significantly overexpression in GBM.
